# Predictors for the incidence of pneumonia among HIV-infected children on antiretroviral therapy in Amhara Regional State Comprehensive Specialized Hospitals, Ethiopia: a multicenter retrospective follow-up study

**DOI:** 10.1186/s13052-024-01695-w

**Published:** 2024-09-04

**Authors:** Gebrehiwot Berie Mekonnen, Fikadie Dagnew Baye, Gashaw Kerebeh, Mengistu Melak Fekadie, Yohannes Tesfahun Kassie, Tiruye Azene Demile, Alamirew Enyew Belay, Asnake Gashaw Belayneh, Bruck Tesfaye Legesse, Wubet Tazeb Wondie, Mengistu Abebe Messelu

**Affiliations:** 1 Department of Pediatrics and Child Health Nursing, College of Health Sciences, Debre Tabor University, P.O. Box: 272, Debre Tabor, Ethiopia; 2 Department of Emergency and Critical Care Nursing, College of Health Sciences, Debre Tabor University, Debre Tabor, Ethiopia; 3 Department of Surgical Nursing, School of Nursing, College of Medicine and Health Sciences, University of Gondar, Gondar, Ethiopia; 4 Department of Adult Health Nursing, School of Health Science, College of Medicine and Health Science, Bahir Dar University, Bahir Dar, Ethiopia; 5 Department of Emergency and Critical Care Nursing, College of Medicine Health Science, Bahir Dar University, Bahir Dar, Ethiopia; 6 Department of Pediatrics and Neonatal Nursing, School of Nursing and Midwifery, Institutes of Health Sciences, Wollaga University, Nekemte, Ethiopia; 7 Department of Pediatrics and Child Health Nursing, College of Medicine and Health Sciences, Ambo University, Ambo, Ethiopia; 8 Department of Nursing, College of Medicine and Health Sciences, Debre Markos University, Debre Markos, Ethiopia

**Keywords:** Antiretroviral therapy, HIV-infected children, Incidence, Pneumonia, Predictors, Ethiopia

## Abstract

**Background:**

Human Immune deficiency Virus (HIV) infected children are at higher risk of developing pneumonia. Particularly, in the early phase of HIV infection, the risk of acquiring pneumonia is high, and it remains a major public health problem even after the test and treatment strategy. There is no clear evidence of the overall incidence of pneumonia among HIV-infected children in Amhara region. Aimed to assess the incidence of pneumonia and its predictors among HIV-infected children receiving Antiretroviral therapy in Amhara Region Comprehensive Specialized Hospitals, 2022.

**Methods:**

A multicenter retrospective follow-up study was conducted from June 10, 2014, to February 28, 2022, among 430 HIV-positive children receiving antiretroviral therapy. A simple random sampling technique was used. The data was taken from the national antiretroviral intake and follow-up forms. The data were collected via the KoBo toolbox and analyzed using Stata version 17. The Kaplan–Meier curve and log-rank test were employed. Bivariable and multivariable Cox regression was carried out to identify predictors of pneumonia and a *P*-value < 0.05 was considered significant in to multivariable analysis.

**Results:**

A total of 407 children with a record completeness rate of 94.7% were analyzed in the study. The incidence rate of pneumonia was 4.55 (95% CI; 3.5, 5.92) per 100 person-years observation. The mean survival time was 77.67 months and the total times at risk during follow-up period were yielding 1229.33 person-year observations. Having CD4 cell count below threshold [AHR; 2.71 (95% CI: 1.37, 5.35)], WHO stage III and IV [AHR: 2.17 (95% CI: 1.15, 4.08)], ever had fair and poor treatment adherence [AHR: 2.66 (95% CI: 1.45, 4.89)], and not initiated antiretroviral therapy within seven days [AHR: 2.35 (95% CI: 1.15, 4.78)] were the positive predictors for incidence of Pneumonia.

**Conclusions:**

In this study, the incidence of pneumonia was lower than the previous studies. CD4 cells below the threshold, ever had fair and poor adherence to antiretroviral therapy, WHO stage III and IV, and not initiated antiretroviral therapy within seven days were significant predictors. Therefore,, it is crucial to detect baseline assessment and give attention to those identified predictors promptly, and timely initiation of antiretroviral therapy need special attention.

## Introduction

Human Immune deficiency Virus (HIV) infected children are at higher risk of developing Pneumonia [1]. Particularly, the risk of developing pneumonia is higher in the early phase of HIV infection [2], and it remains a major public health problem even after test and treat strategy. The underlying HIV infection is a major risk factor for Pneumonia morbidity and mortality [3], those infected children’s are 6.5 times at higher risk of developing pneumonia when compared with those uninfected [4].

Globally, only 1% of pneumonia infections and 9% of pneumonia deaths are attributed to HIV/AIDS, However, in Africa 3% of pneumonia infections and 17% of pneumonia death among children are attributable to HIV/ AIDS [3]. In USA, the incidence of pneumonia was 21.5 per 1000 PY [5], and 33.2 per 1000 person year observation (PYO), and the incidence is particularly higher among under-five children [6].

Pneumonia accounts 25.01% of opportunistic infections among HIV-infected children on antiretroviral therapy (ART) in Low- and Middle-Income Countries (LMIC) including Ethiopia [7]. In low-income countries, 1.4 million cases of pneumonia occurred among HIV-infected children. In Africa, 1.29 million cases of pneumonia have occurred among HIV infected children [8].

The incidence of pneumonia among HIV-infected children varies among different countries and setting, in USA 3.32 per 100 PYO [9], in Sub-Saharan countries it ranges from 12.2 to 26.5 PYO [4], in East Africa 3.9 to 20 PYO [10], while in our country Ethiopia the incidence of pneumonia in Bahir Dar public Health institutions and University of Gondar Comprehensive Specialized Hospital were 5.57 [11] and 13.5 per 100 PYO [12] respectively.

Despite variation in studies, the predictors of pneumonia among HIV-infected children in US, India, in Sub Saharan countries, East Africa, and in Ethiopia underweight, WHO clinical staging), high viral load, cotrimoxazole preventive therapy(CPT), adherence level, anemia, low CD4 count, young age, and tuberculosis preventive therapy (TPT) were significant variables [4, 9–15], due to geographical and implementation variation additional investigation is necessary.

To reduce pneumonia and other HIV-associated opportunistic infections, different strategies and interventions such as reduction of exposure, chemoprophylaxis, immunization, and immediate HAART after testing since 2014 have been done [2]. In addition, Ethiopia has Health Sector Transformation Plan II (HSTP-II) which aims to reduce HIV/AIDS and its complications by the end of 2025 [16].

Even though different interventions are undertaken, still pneumonia causes significant morbidity and mortality, and remains the common opportunistic infection among HIV-infected children on ART, and also there is limited evidence on the overall incidence of pneumonia and its predictor in the study area after test and treat strategy. Hence, in this study, rapid initiation of ART was incorporated as an independent predictor for the incidence of pneumonia. Furthermore, necessitating ongoing efforts to provide updated information for effective test and treat strategies.

This finding helps to increase the existing knowledge of health professionals and to provide input for program planners and decision-makers on HIV/AIDS care and support at regional as well as national level in achieving the above strategic plans. Therefore, this study aimed to assess the incidence of pneumonia and its predictors among HIV-infected children on ART in Amhara Regional State Comprehensive Specialized Hospitals, Ethiopia, 2022.

## Methods and materials

### Study design and period, study area

A multicenter institution-based retrospective follow-up study was conducted from June 10, 2014, to February 28, 2022 in Amhara region comprehensive specialized Hospitals. The University of Gondar, Felege-Hiwot, Debre-Markos, Debre Tabor, Dessie, Woldia, and Debre Birhan were the seven Amhara Regional State Comprehensive Specialized Hospitals participating, except Tibebe Ghion due to inadequate study population, and recently established specialized comprehensive specialized. With an estimated area of 159,173.66 square kilometers, the Amhara Region is situated in Ethiopia's northwest, northeast, and north-central regions. The Region is divided into 183 Woreda, three city administrations, and twelve zones for administrative purposes.

The Ethiopian Demographic and Health (EDH) report from January 2022 projects the region's total population to be 30,848,988. Eight of the 81 hospitals in the region are comprehensive, specialized teaching hospitals, and there are 3560 health posts in addition to 858 health centers, according to the Amhara National Regional Health Bureau's annual performance report. Specialized units for ART care and accompanying services are available at these hospitals [17]. As part of the National AIDS Control Program, the Amhara Comprehensive Specialized Hospitals have been offering free ART services since 2005. From June 10, 2014, to February 28, 2022, 928 HIV-positive children were newly enrolled on antiretroviral therapy (ART).

### Study participants

The source populations for this study were all HIV-infected children receiving ART in Amhara Regional State Comprehensive Specialized Hospitals, with the study population including those HIV-infected started on ART from June 10, 2014 to February 28, 2022. All newly enrolled HIV-infected children who have been on ART at Amhara Regional State Comprehensive Specialized Hospitals during the study period included in this study. Records with incomplete baseline information (CD4 count, hemoglobin level, weight, and height) and unknown date of ART initiation and outcome status were excluded from this study, and children who already develop pneumonia were excluded from this study [14, 15].

### Sample size determination and sampling procedures

With the following presumptions the first objective, the sample size was calculated using the single population proportion formula: previous study conducted in Bahir Dar public health institutions the cumulative incidence of pneumonia among children on ART was 20.47% [18], with a 95% confidence interval and a 4% margin of error. The calculated sample sizes were 430, which was the final sample size for this study after adding 10% for data incompleteness rate.

For the second objective, the sample size was calculated on the basis of common significant predictor variables using Cox models, implemented in STATA version 17 (Table 1).

**Table 1:** Sample size determination based on predictors of incidence of pneumonia among HIV-infected children receiving ART, using cox-model in STATA version 17

Variables	Assumptions				
	Power	Hazard Ratio (HR)	Probability of event	Probability of Withdraw	Sample size (n)
Weight for age(WFA)	80	2.62	0.2047	0.1	141
WHO clinical staging	80	2.8	0.2047	0.1	184
Cotrimoxazole preventive therapy (CPT)	80	3.01	0.2047	0.1	161

Therefore, the final sample size for this study was 430

The sample was allocated proportionally to each Comprehensive Specialized Hospitals based on the number of HIV infected children on ART stated as follows: 108 out of 233 from University of Gondar, 96 out of 207 from Felege Hiwot, 60 out of 127 from Debre Markos, 45 out of 97 from Debre Tabor, 55 out of 119 from Dessie, 36 out of 78 from Woldia, and 30 out of 65 from Debre Birhan. Records were selected using simple random techniques.

### Variables of the study

The dependent variable for this study was the occurrence of pneumonia during the follow-up period. The independent variables included: Socio-demographic characteristics (i.e., age, sex, residence, current parent's status, educational status of the caregiver, and marital status of the caregiver); Baseline clinical, nutritional, and laboratory characteristics (i.e., CD4 count, hemoglobin level, anthropometric indices, HIV disclosure status, functional& developmental status); ART and medication-related characteristics (i.e., Baseline ART regimen, presence of regimen change, level of ART adherence, Tuberculosis preventive therapy, Cotrimoxazole preventive therapy (CPT), ART side effect, Initiation of ART within seven days) [19].

### Operational definition

**Children:** Individuals with ages less than 15 years old [15, 19–21].

**Pneumonia cases** were labeled when HIV-infected children pneumonia developed in the first time after ART initiation during the follow-up period and as documented by the ART health personnel [2].

**An event** was labeled when HIV-infected children developed pneumonia after ART initiation during the follow-up period.

**Censored**: Children, who were lost to follow-up, dropped out, formally transferred out after initiating ART, died due to any causes, or did not develop the events until the last visit.

**Lost to follow up (LTFU):** was recorded when HIV-infected children missed their appointments from one month to three months [19].

A child is considered as stunting when the Height for Age (HFA) or length for age (LFA) Z-score less than -2 SD [2], and **Wasting is considered when** the weight for height (WFH) Z-score is less than -2 SD for less than five years, or if Body mass index (BMI) for age Z-score is less than -2 SD for greater than five years [2].

**The level of adherence to ART:** Good adherence is reported with compliance equal to or greater than 95% or ≤ 3 missed doses per month as documented by the ART health personnel; fair reflected 85–94% compliance and between 4 and 8 missing doses per month) as documented by the ART health personnel, and poorly reflected less than 85% compliance or ≥ 9 missed dose per month) as documented by the ART health personnel [19].

**Child developmental status** was classified as appropriate (able to attain milestones for age), delayed (failure to attain milestones for age); and regression (loss of what has been attained for age) as documented by the ART health personnel [2].

**Rapid initiation of ART:** ART initiation care and support on the same day of HIV confirmation or within seven days [23].

A child is considered as Anemic (low hemoglobin level) when the hemoglobin level of below 10 mg/dl [14, 15, 18, 20]. **CD4 counts or percentage (%) below the threshold** is considered if the child had CD4 cell counts < 1500/ mm3 or 25% for age < 12 months, CD4 cell counts < 750/ mm3 or < 20% for age 12–35 months, CD4 cell counts < 350/mm3 or < 15% for age 36–59 months, and CD4 cell counts < 200/mm3 or < 15% for age ≥ 60 months [2].

**Baseline data:** Any laboratory tests obtained at the time of ART initiation were considered baseline data. However, if laboratory tests were not done during ART initiation, any laboratory tests were done within a month of ART initiation [24].

### Data collection tool, procedures, and quality control

The data were collected from the ART intake form, follow-up form, and children`s charts using the data extraction tool adopted from Ethiopian ART guidelines [2]. The data extraction tool consists of socio-demographic, clinical, laboratory, ART, and medication-related variables. Data were collected by seven bachelor's degree nurses who had smartphones and three supervisors who were familiar with the ART follow-up and taking basic ART training. The data extraction tool was pretested 5% of the sample size two weeks before the actual data collection period at University of Gondar Comprehensive Specialized Hospital. Moreover, one-day onsite training was given on how to review ART follow-up and medical records, data collection methods, and the objective of the study for data collectors and supervisors. Data were collected using the KoBo toolbox software with online server which was prepared with relevant restrictions by trained nurses working in the Hospitals.

### Data processing and analysis

The KoBo toolbox was utilized to gather data, which were then exported to STATA version 17(MP) statistical software programs for examination. Anthropometric indices were also produced using the WHO anthro and WHO anthroPlus software. To present the results, descriptive statistics were performed using the mean, rate, frequency, percentage, tables, and figures. An analysis of multi-collinearity was used to determine whether the predictor variables were related.

The Kaplan–Meier failure function and a log-rank test was used to estimate pneumonia-free survival probabilities, and to compare different categorical predictor variables. The Cox proportional hazard (ph) model assumption was checked for variables in the Log–log plot (graphically), and the final model was evaluated using the Schoenfeld residual (Global) test (*p* = 0.804). At this time, all assumptions were fulfilled. The Cox-Snell residual test was used to check the goodness of fit. The Multivariable Cox proportional hazards model was used to identify predictors. A 95% confidence interval (CI) for AHR was estimated to see the strength of association. A *P*-value ≤ 0.05 was used to declare the statistical significance.

## Results

### Baseline socio-demographic, clinical, nutritional and laboratory characteristics of HIV-infected children on ART

A total of 407 HIV-infected children’s medical records with a completeness rate of 94.65% were included in this study. The mean (± SD) ages of children were 7.56 ± 4.16 years. Twenty-eight percent of the children were in the age group of < 5 years. The majority (78.4%) of children had a CD4 cell count above the threshold; and based on functional status 71.8% were working and appropriate motor developmental status were 69.8%. About 16.2% had anemia; 29.98% and 49.6% nutritional status were wasted and stunted respectively (Table 2).

**Table 2:** Baseline socio-demographic, clinical, nutritional and laboratory characteristics of HIV-infected children on ART at Amhara Regional State Comprehensive Specialized Hospitals, Ethiopia, 2022 (*n* = 407)

Characteristics	Outcome status		Total (n)	IR per 100 PY
	**Event (%)**	**Censored (%)**		
**Age (in years)**				
< 5	22 (19.30)	92 (80.70)	114(28.0)	7.33
5–9	22 (15.71)	118 (84.29)	140(34.4)	4.59
10–14	12 (7.84)	141 (92.16)	153(37.6)	2.67
**Sex**				
Female	24(14.29)	144(85.71)	168(41.3)	4.66
Male	32(13.39)	207(86.61)	239(58.7)	4.48
**Residence**				
Rural	14 (11.57)	107(88.43)	121(29.7)	4.07
Urban	42(14.69)	244(85.31)	286 (70.3)	4.74
**Current parent status**				
Both parents alive	35(12.32)	249 (87.68)	284(69.8)	3.89
One or both parent alive	21 (17.07)	102 (82.93)	123(30.2)	6.35
**Educational Status of the care giver**				
No formal education	20 (14.29)	120 (85.71)	140(34.3)	4.95
Primary	16 (12.8)	109 (87.2)	125(30.6)	4.16
Secondary	16 (19.23)	63 (80.77)	79(19.4)	6.7
Tertiary(College& above)	5 (7.81)	59 (92.19)	64(15.7)	2.3
**Marital status of the care giver**				
Married	28 (12.67)	193 (87.33)	221(54.3)	4.11
Unmarried	12 (12.5)	84 (87.5)	96(23.6)	4.41
Divorced	9 (15.52)	49(84.48)	58(14.2)	5.22
Widowed	7(21.88)	25(78.22)	32(7.9)	6.78
**CD4 cell count**				
Above threshold	33(10.34)	286(89.66)	319(78.4)	3.25
Below threshold	23(26.14)	65(73.86)	88(21.6)	10.65
**WHO staging**				
Stage I&II	34(10)	306(90)	340(83.54)	3.24
Stage III&IV	22(32.84)	45(67.6)	67(16.46)	12.19
**Anemia**				
No	39(11.44)	302(88.56)	341(83.8)	3.71
Yes	17(25.76)	49(74.24)	66(16.2)	9.49
**Functional status**				
Working	23(11)	186(89)	209(71.8)	3.41
Ambulatory	8(10.13)	71(8987)	79(27.1)	1.48
Bedridden	0(0)	3(100)	3(1.1)	-
**Developmental status**				
Appropriate	20(24.69)	61(75.3)	81(69.8)	10.29
Delayed	5(16.13)	26(83.87)	31(26.7)	6.77
Regressed	0(0)	4(100)	4(3.44)	-
**Weight for height Z score**				
Normal	38(13.33)	247(86.67)	285(70)	4.21
Moderate	11(25.58)	32(74.42)	43(10.6)	10.84
Severity	7(8.86)	72(91.14)	79(19.4)	3.09
**Height for age Z score**				
Normal	31(15.12)	174(84.88)	205(50.4)	4.58
Moderate	8(10.81)	66(89.19)	74(18.2)	3.57
Severity	17(13.28)	111(86.72)	128(31.4)	5.17

### ART and medication-related characteristics

The proportion of good adherence to ART, intake of TB preventive therapy and Cotri-moxazole preventive therapy during the follow-up period by the children was 68.3%, 65.4% and 81.6%, respectively. In addition, only 41.8% of the study participants were of them initiated ART within seven days after admission (Table 3).

**Table 3:** Baseline ART and medication-related characteristics of HIV-infected children on ART in the Amhara Regional State Comprehensive Specialized Hospitals, Ethiopia, 2022 (*n* = 407)

Characteristics	Event (%)	Censored (%)	Total (n)	IR per 100 PY
**ART drug adherence level**				
Good	24 (8.63)	254 (91.37)	278(68.3)	2.66
Fair or poor	32 (24.81)	97(75.19)	129(31.7)	9.8
**Cotri-moxazole Preventive Therapy taken**				
Yes	45 (13.55)	287 (86.45)	332(81.6)	4.31
No	11 (14.67)	64 (85.33)	75(18.4)	5.89
**ART side effect**				
No	37(13.7)	233(86.3)	270(66.3)	4.7
Yes	19(13.87)	118(86.13)	137(33.7)	4.3
**Presence regimen change**				
Yes	32(16.33)	164(86.13)	137(33.6)	4.79
No	24(11.37)	187(88.63)	211(51.8)	4.27
**Rapid initiation of ART**				
Yes	10(5.88)	160(94.12)	170(41.8)	2.06
No	46(19.41)	191(80.59)	237(58.2)	6.17
**TB Preventive Therapy taken**				
Yes	28(10.53)	238(89.47)	266(65.4)	3.23
No	28(19.86)	113(80.14)	141(34.6)	7.73

### Incidence of pneumonia during the follow-up period

Four hundred and seven HIV-infected children on ART had a follow-up time from 1 to 92 months with a total time at risk of 14758 person-months or 1229.33 person-years observation. The mean time of observations was 36.26 months. The overall pneumonia incidence was 4.55 (95%CI; 3.5, 5.92) per 100 PY observations. The mean survival time was 77.67 with (95% CI: 74.33, 81.02) months.

### Kaplan–Meier failure function

**Fig. 1:**
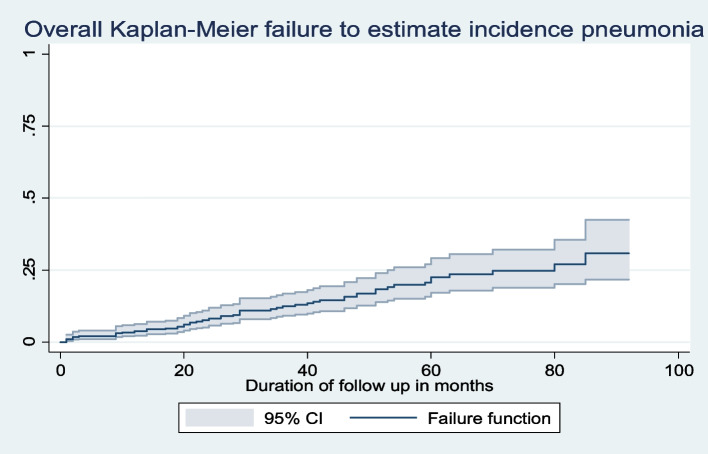
Overall Kaplan–Meier failure to estimate incidence of pneumonia probability among HIV-infected children on ART in Amhara Regional State Comprehensive Specialized Hospitals, Ethiopia, 2022

The probability of acquiring pneumonia for the total follow up time by the end of follow-up was 0.304 (95% CI; 0.2211, 0.4088); whereas it was 0.0207 (95% CI; 0.0104, 0.041), 0.035 (95%CI; 0.0205, 0.0596), 0.1207 (95% CI; 0.0879, 0.1644), and 0.2504 (95% CI; 0.1919, 3230) at the end of 6 months, 1 year, 3 years, 5 years, and 7 years respectively (Fig. 1).

### Proportional hazard assumption

**Fig. 2:**
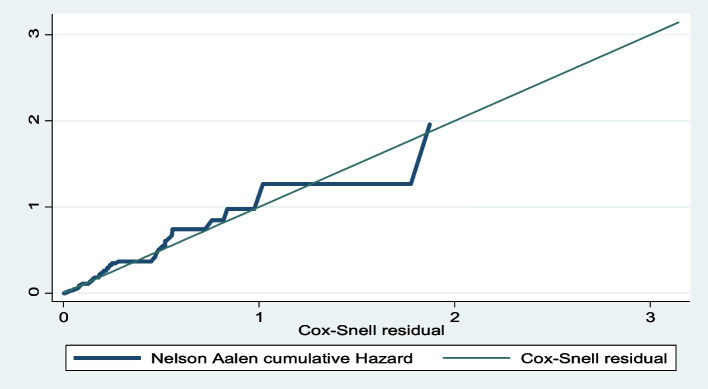
The goodness of fit test for the Cox-proportional hazard regression model

Based on the proportional hazard assumption test using Schoenfeld residual, and log–log plot (graphically) all of the covariates fulfilled the assumption and the overall global model satisfies the proportional hazard assumption (global test, *p* = 0.804). The Cox-Snell residual test was used to check the goodness of fit. A residual is a difference between an observed data point and a predicted or fitted value and as the following graph indicates the cox regression model fits with the Cox-Snell residual and the predicted hazard at 45^0^ (Fig. 2).

### Predictors for incidence of pneumonia among HIV-infected children on ART

In the bi-variable Cox regression analysis, age, status of parents, educational status, initiation of ART within seven days, CD4, WHO staging, anemia, weight for height Z score, ART adherence, and CPT were candidates for multivariable analysis. However, in the multivariable Cox regression analysis, CD4 cell count, ART adherence level, WHO clinical staging, and initiation of ART within seven days of admission were significant predictors for the incidence of pneumonia among HIV-infected children on ART.

The risk of developing pneumonia among children presented with a CD4 cell count below the threshold was 2.71 times [AHR: 2.71 (95% CI: 1.37, 5.35)], higher as compared to those children with a CD4 count above the threshold.

The probability of developing pneumonia among children with advanced stage (WHO stage III and IV) is 2.17 times [AHR: 2.17 (95% CI: 1.15, 4.08)] higher as compared to WHO stage I and II children.

HIV-infected children on ART who had fair and poor ART adherence were 2.66 times [AHR: 2.66 (95% CI: 1.45, 4.89)] increase the risk to develop pneumonia as compared to those children who had good ART adherence.

Furthermore, the risk of developing pneumonia among HIV-infected children who did not initiate ART within seven days of admission was 2.35 times [AHR: 2.35 (95% CI: 1.15, 4.78)] higher as compared to those who had initiated ART at or immediately after diagnosis (Table 4).

**Table 4:** Bi-variable and multivariable Cox-proportional hazard analysis of predictors of incidence of pneumonia among HIV-infected children on ART in Amhara Regional State Comprehensive Specialized Hospitals, Ethiopia, 2022 (*n* = 407)

Predictors	Status of Pneumonia		CHR (95% CI)	AHR (CI)
	Event (*n* = 56)	Censored (*n* = 351)		
**Age in years**				
< 5 years	22	92	2.78(1.38, 5.64)	1.03(0.42, 2.54)
5 to 9 years	22	118	1.71(0.84, 3.45)	1.57(0.74, 3.33)
10 to 14 years	12	141	**1**	**1**
**Status of the parents**				
Both alive	35	249	**1**	**1**
One or both deceased	21	102	1.63(0.93, 2.81)	1.19(0.64, 2.22)
**Educational status of the care giver**				
No formal education	20	120	2.22(0.83, 5.92)	2.22(0.83, 5.92)
Primary	16	109	1.83(0.67, 5.01)	1.83(0.67, 5.01)
Secondary	16	63	2.99(1.09, 8.23)	1.75(0.58, 5.25)
Tertiary	5	59	1	1
**Initiated Antiretroviral therapy within seven days**				
Yes	10	160	1	1
** No**	46	191	**2.96(1.49, 5.87)**	**2.35(1.15, 4.78)**^**a**^
**CD4 count threshold**				
Above	33	286	1	1
** Below**	23	65	**3.32(1.94, 5.66)**	**2.71(1.37, 5.35)**^**b**^
**WHO staging**				
Stage I&II	34	306	1	1
Stage III&IV	22	45	**3.73(2.18, 6.38)**	**2.17(1.15, 4.08)**^**a**^
**Anemia status**				
No	39	302	1	1
** Yes**	17	49	2.57(1.45, 4.55)	1.03(0.51, 2.07)
**Weight for height Z score**				
Normal	38	247	1	
Moderate	11	32	2.67(1.35, 5.28)	1.83(0.87,3.82)
Severity	7	72	0.74(0.33, 1.66)	0.68(0.28, 1.68)
**ART adherence level**				
Good	24	254	1	1
Fair or poor	32	97	**3.81(2.24, 6.49)**	**2.66(1.45, 4.89)**^**b**^
**Ever taking cotri-moxazole preventive therapy**				
Yes	28	238	1	1
No	28	113	2.47(1.45, 4.19)	1.21(0.64, 2.28)

AHR = Adjusted Hazard Ratio

CHR = Crude Hazard Ratio

1, reference

^a^Significant at < 0.05

^b^Significant at < 0.01

## Discussion

Pneumonia on HIV-infected children on ART is the predominant cause of morbidity and mortality among opportunistic infections. In this regard, our study aimed to assess the incidence and predictors of pneumonia among HIV-infected children on ART in Amhara Regional State Comprehensive Specialized Hospitals, Ethiopia, 2022.

The overall incidence of pneumonia among HIV-infected children in this study was 4.55 (95% CI; 3.5, 5.92) per 100 Person-years of observations (PYO), this finding was consistent with the study done in Northern India [25] and Bahir Dar city public Hospitals [11]. However, it was higher than the studies from the United States (3.32 per 100 person-years) [9], and Europe (0.54 per 100 person-years) [26]. The possible explanations for this difference might be due to developed countries have better diagnosis and management technologies so then the life long of the pneumonia will short due to appropriate care and support. And also, it might be due to clinical characteristics of the current study, one-third of the study participant have no formal education and unfavorable ART drug adherence.

However, this finding is lower than the studies conducted in Spain (13.7 per 100 child-years) [27] and Latin America (8.1 per 100 person-years) [28]. Difference might or be due to participant's variation for example in Spain HIV-infected children less than 17 years of age and had a larger sample size than the current study, and the reason for the difference might be related to the study population; The duration and longitudinal nature of the study may be the cause of the discrepancy with the study conducted in Latin America. Additionally, the present study, well-organized HIV care and support were given after new strategy, treatment, and care for HIV-infected children may have an impact, as may the implementation of new guidelines that use a test-and-treat strategy [22].

In the present study, CD4 cell count below the threshold, poor and fair ART adherence level, WHO stage III&IV, and ever not initiating antiretroviral therapy within seven days were predictors.

Children presented with a CD4 cell count below the threshold were increased 2.71 times probability to develop pneumonia as compared to their counterparts. This finding was supported by studies conducted in Latin America [28], India [25], the USA [29], Northern Ethiopia [30], and Debre Markos, Ethiopia [31]. This is due to white blood cells called CD4 cells aid in the activation of other white blood cells in the immune system when being with CD4 count below threshold is the very indicative of low immunity and increase susceptibility for opportunistic infections such as pneumonia [32].

The probability of developing pneumonia in WHO clinical stage III and IV 2.17 times increasing the risk as compare to WHO clinical stage I and III. This finding is supported by different studies conducted in India [33], Asia [34], Nigeria [35], and Ethiopia [36]. This is due to those children being in clinical stage III&IV which is highly indicative for very low immunity level and it favors for opportunistic infection among these pneumonia is very common [37, 38].

HIV-infected children on ART who had fair and poor ART adherence were 2.66 times increase risk of developing pneumonia as compared to those children who had good ART adherence. This result is consistent with the studies conducted in Debre Markos, Ethiopia [31], Debre Tabor & University of Gondar [18], and Bahir Dar Public Hospitals [39], Brazil [40], and India [41]. The possible reason is due to the rapid initiation of ART without intensive counseling and inadequate preparation to accept ART leads to poor and fair drug adherence. Being poor and fair adherent to the ART medications directly means that, the ART increased the viral replication to the maximum, healthy and improves the quality of life compromised, the risk of developing pneumonia is maximized, and the risk of drug resistance is increased [2, 23, 42].

Children with HIV who were on ART and had not started treatment within seven days were 2.35 times increasing the probability of pneumonia than children who had started treatment within seven days of admission or enrollment. This might be due to the early initiation of ART to reduce delays, enhances medication uptake and improve viral suppression rates in children with HIV, increase HIV retention care, viral suppression, and reduce HIV transmission [43]. Hence, pneumonia incidence will be decreased when ART is commenced swiftly, unless the children had not started ART quickly [32]. Starting ART early could be beneficial in meeting the goal of 95% of patients obtaining viral suppression by 2025, which would help lower the rate of pneumonia.

### Limitations of the study

As a limitation, since the data were collected retrospectively, the study relied on the already available and recorded information on the type of diseases during follow-up that missed important variables. The incidence of pneumonia might be underestimated due to limited capacity to make definitive diagnosis for asymptomatic cases.

## Conclusion

In this study, the incidence of pneumonia was low compared to the previous studies. Baseline clinical variables such as having CD4 cellacount below the threshold, ever having fair and poor adherence toART drug adherence, advanced HIV AIDS, and lately ART initiation were significant predictors of the incidence of pneumonia among HIV-infected children on ART.

### Recommendation

The Federal Ministry of Health, the Amhara National Regional Health Bureau, Comprehensive Specialized Hospitals, and health care providers shall strengthen their roles to reduce the incidence of pneumonia among children on ART relatively reduces comparing from the previous studies. And shall by critically screening, monitoring, and treating or managing significant predictor variables such as baseline CD4 cell count below the threshold, poor and fair adherence level, WHO clinical staging, enhance rapid ART initiation in each comprehensive specialized hospital. The researchers shall be conduct qualitative research, and further prospective cohort studies shall be conducted by incorporating important predictors like income status, caregivers' occupational status, family size, and viral load.

## Data Availability

Data will be provided by the associated author upon reasonable request.

## Abbreviations

AHR: Adjusted Hazard Ratio
AIDS: Acquired Immune Deficiency Syndrome
ART: Antiretroviral Therapy
BMI: Body Mass Index
CPT: Cotri-moxazole Preventive Therapy
CD4: Cluster of Differentiation 4
CSHs: Comprehensive Specialized Hospitals
HAART: Highly Active Antiretroviral Therapy
HFA: Height -For –Age
HIV: Human Immunodeficiency Virus
HSTP-II: Health Sector Transformation plan-II
LFA: Length -For –Age
PLHIV: People Living with Human Immunodeficiency Virus
PYO: Person-Year-Observations
SD: Standard Deviation
SDG: Sustainable Development Goals
SSA: Sub- Saharan Africa
TB: Tuberculosis
UoG: University of Gondar
WFH: Weight -For –Height
WHO: World Health Organization
